# Distinct safety profiles of liposomal and conventional irinotecan: insights from clinical experience and real-world data

**DOI:** 10.3389/fphar.2026.1745317

**Published:** 2026-04-21

**Authors:** Han Shan, Shuohan Huang, Qiong Du, Mengmeng Wang

**Affiliations:** 1 Department of Pharmacy, Fudan University Shanghai Cancer Center, Shanghai, China; 2 Department of Oncology, Shanghai Medical College, Fudan University, Shanghai, China

**Keywords:** adverse events, drug safety, liposomal irinotecan, non-liposomal irinotecan, pharmacokinetics

## Abstract

**Background:**

Liposomal irinotecan (nal-IRI) and conventional irinotecan (IRI) represent two formulations with distinct pharmacokinetic properties, yet their comparative safety profiles remain incompletely characterized. This study aimed to systematically evaluate their adverse event (AE) patterns through integrated pharmacovigilance and clinical analyses.

**Methods:**

We conducted a dual-approach investigation combining: (1) a single-center retrospective study of 308 pancreatic cancer patients (nal-IRI = 131; IRI = 177) treated between December 2023-September 2024, and (2) disproportionality analysis of FAERS database reports (2004–2024) using reporting odds ratios (RORs) and proportional reporting ratios (PRRs).

**Results:**

The retrospective study demonstrated significantly higher incidence of grade 3–4 leukopenia with nal-IRI (9.16% vs. 3.39%, p < 0.05) and earlier onset of hematologic/hepatic toxicities (median time difference: 20–30 days, p < 0.05). No significant differences were observed in non-hematologic toxicities between groups. FAERS analysis revealed distinct AE patterns: nal-IRI reports were predominantly associated with fatal outcomes (43.76%) and cholangitis (PRR = 996.70) in pancreatic cancer patients, while IRI showed conventional chemotherapy toxicities in colorectal cancer patients.

**Conclusion:**

These findings suggest that nal-IRI and IRI exhibit distinct safety profiles, which are partly attributable to differences in their liposomal pharmacokinetics, but also reflect the underlying disease characteristics and prognostic profiles of their respective patient populations. These findings emphasize the need for enhanced monitoring during initial nal-IRI treatment cycles and suggest differential safety management strategies for the two formulations. Future multicenter studies with pharmacokinetic assessments are warranted to further elucidate these differences.

## 1 Introduction

Irinotecan (IRI), a potent topoisomerase I inhibitor, has established itself as a cornerstone in the treatment of various malignancies, particularly metastatic colorectal cancer and pancreatic cancer (Voutsadakis et al., 2024). Despite its clinical efficacy, the therapeutic utility of conventional IRI is frequently compromised by dose-limiting toxicities, notably myelosuppression and delayed-onset diarrhea (De With et al., 2023). To address these limitations, liposomal irinotecan (nal-IRI) was developed, employing nanocarrier technology to achieve targeted drug delivery and sustained release properties, thereby theoretically enhancing therapeutic efficacy while mitigating systemic toxicity (Milano et al., 2022). Nevertheless, a comprehensive evaluation comparing the safety profiles of these two formulations in real-world clinical settings remains lacking.

The current understanding of safety differences between liposomal and conventional IRI remains contentious. On one hand, the liposomal formulation demonstrates superior biodistribution characteristics, exhibiting preferential tumor targeting through enhanced permeability and retention effects, which may reduce systemic exposure to free drug and consequently diminish certain class-associated toxicities (Fra and mpton, 2020). Conversely, the liposomal encapsulation protects the drug from premature plasma metabolism, resulting in prolonged systemic circulation and enhanced cytotoxic potential compared to its non-liposomal counterpart (Milano et al., 2022). These distinct pharmacokinetic properties may introduce novel safety concerns, including drug accumulation-related hepatobiliary adverse effects (Brendel et al., 2021). Furthermore, the differential indication spectra between the two formulations - with nal-IRI primarily employed in pancreatic cancer and conventional IRI more commonly used in colorectal cancer - adds another layer of complexity to safety assessments.

To address these knowledge gaps, our study employs a multifaceted analytical approach: First, we perform a single-center, real-world retrospective study focusing on the incidence and temporal patterns of hematologic and hepatic toxicities. Subsequently, we conduct a large-scale pharmacovigilance study utilizing the US Food and Drug Administration Adverse Event Reporting System (FAERS) database to identify differential safety signals between the two formulations. By integrating pharmacoepidemiologic data with clinical observations, this investigation aims to provide evidence-based guidance for optimal formulation selection. The findings will not only have immediate clinical implications but also contribute methodological insights for comparative safety evaluations between nanomedicines and conventional chemotherapeutic agents.

## 2 Methods

### 2.1 Screening of patients in our center

The real-world clinical data in this study were obtained from Fudan University Shanghai Cancer Center. Patient inclusion criteria consisted of: (Voutsadakis et al., 2024): histologically confirmed pancreatic cancer diagnosis; and (De With et al., 2023) treatment with either nal-IRI or conventional IRI between 1 December 2023 and 1 September 2024. Patients with incomplete medical records were excluded from the analysis.

**FIGURE 1:**
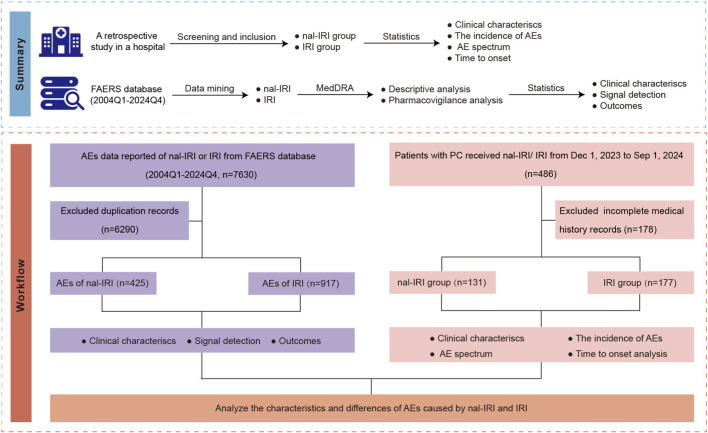
Flowchart visualizing the sequential analytical procedures adopted in this study. Abbreviations: AE, adverse event; FAERS,US Food and Drug Administration Adverse Event Reporting System; IRI, non-liposomal irinotecan; MedDRA, Medical Dictionary for Regulatory Activities; nal-IRI, nanoliposomal irinotecan.

Eligible patients were stratified into two cohorts based on their treatment regimen: the nal-IRI group and the conventional IRI group. Comprehensive clinical data were extracted from electronic medical records, including demographic characteristics (age and gender), disease stage at treatment initiation, concomitant chemotherapy regimens, dosage and treatment duration of the target drugs, detailed history of prior chemotherapy exposure, and detailed AE profiles (Figure 1).

This retrospective study was conducted in full compliance with ethical standards and was approved by the Independent Ethics Committee of Fudan University Shanghai Cancer Center. All patient data were anonymized and handled in accordance with institutional guidelines for the protection of human subjects.

### 2.2 FAERS database data source and search strategy

For the pharmacovigilance analysis in this study, we extracted publicly available adverse event reports from the FAERS database. Data retrieval and processing were performed using OpenVigil 2.1, a validated pharmacovigilance analysis tool (McIntyre and Kwan, 2024) that automatically eliminates duplicate reports and handles missing data. Our study focused on two formulations of IRI: nai-IRI and conventional IRI. Due to the lack of distinct drug name identifiers for these two formulations in the FAERS “Drug name” field, we employed the “Pharma product” field for precise identification. Specifically, reports containing “onivyde” (the brand name for nal-IRI) were classified as nal-IRI cases, while those containing “camptosar” (the brand name for conventional IRI) were categorized as IRI cases. All included reports were restricted to those where the study drugs were designated as “Primary Suspect Drug” in the “Role code” field.

We collected all relevant adverse event (AE) reports from the first quarter of 2004 through the fourth quarter of 2024. To standardize the data, we utilized the Medical Dictionary for Regulatory Activities (MedDRA, version 25.1) classification system. AEs were systematically categorized using both the Preferred Term (PT) for specific event identification and the System Organ Class (SOC) for broader organ system classification (Figure 1). This dual-level classification approach enabled comprehensive analysis of both specific AEs and their broader systemic manifestations.

### 2.3 Statistical analysis

In the single-center real-world study, categorical variables were analyzed using Fisher’s exact test or Pearson’s χ^2^ test as appropriate. Continuous variables were presented as medians with interquartile ranges and compared using the Wilcoxon rank-sum test. The median time-to-onset of AEs was evaluated through Kaplan-Meier analysis with log-rank tests for between-group comparisons. For clinical outcome analyses, we applied Bonferroni correction to adjust for multiple comparisons, setting the significance threshold at α = 0.01 (0.05/5 comparisons). All other statistical comparisons maintained the conventional significance level of p < 0.05. This multi-tiered analytical approach ensured rigorous evaluation of both safety signals and clinical outcomes while appropriately controlling for type I error.

For the FAERS database analysis, disproportionality analysis was performed using three complementary statistical methods: proportional reporting ratio (PRR), χ^2^ (chi-square), and reporting odds ratios (RORs). A positive safety signal was defined when meeting all the following criteria: (Voutsadakis et al., 2024): ≥3 reported cases of the AE; (De With et al., 2023); lower limit of the 95% confidence interval (CI) for ROR >1; (Milano et al., 2022); PRR ≥2; and (Fra and mpton, 2020) χ^2^ ≥ 4 (p < 0.05). This stringent definition ensured robust signal detection while minimizing false positives.

Statistical analyses and graphical representations were generated using three specialized software platforms: GraphPad Prism (v5.01, Dotmatics) was employed for data visualization and basic statistical testing, while IBM SPSS Statistics (v21.0) and SAS (v9.4) were utilized for comprehensive data analysis. This multi-software approach ensured optimal analytical capabilities for different aspects of our research methodology, with each platform being selected based on its specific strengths in statistical computation or graphical presentation.

## 3 Results

### 3.1 Incidence and time-to-onset of AEs: a single-center retrospective comparison of nal-IRI and IRI

This real-world retrospective study analyzed 308 pancreatic cancer patients treated with either nal-IRI (n = 131) or IRI (n = 177), with a median follow-up of 6.7 months (range 0.9–14.6 months). As shown in Table 1, the demographic and clinical characteristics were balanced between groups, with median ages of 62 years (nal-IRI) and 60 years (IRI), and similar gender distributions (60.31% vs. 58.19% male, respectively). Both groups most frequently received oxaliplatin and fluoropyrimidines as concomitant chemotherapy, with no significant differences in baseline characteristics except for mean dose intensity (p < 0.05). Importantly, the two groups were also comparable with respect to disease stage distribution and prior chemotherapy exposure. No significant differences were observed in the proportion of patients with Stage I–IV disease, nor in the history of exposure to individual chemotherapy agents (all p > 0.05). These findings indicate that the two cohorts were well-balanced in key prognostic factors and prior treatment burden.

**TABLE 1:** Characteristic of patients in the retrospective study at baseline.

Baseline characteristics	Nal-IRI	IRI	p Value
Patients (n)	131	177	​
Age (years)	​	​	0.474
Median	62	60	​
Range	34–78	35–75	​
Gender	​	​	0.709
Male	79 (60.31)	103 (58.19)	​
Female	52 (39.69)	74 (41.81)	​
Concomitant chemotherapy regimens	​	​	0.069
Albumin-bound paclitaxel	6 (4.58)	2 (1.13)	​
Albumin-bound paclitaxeland fluoropyrimidines	7 (5.34)	4 (2.26)	​
Fluoropyrimidines	28 (21.37)	51 (28.81)	​
Oxaliplatin and fluoropyrimidines	90 (68.70)	120 (67.80)	​
Disease stage	​	​	0.245
I	5 (3.82)	12 (6.78)	​
II	26 (19.85)	31 (17.51)	​
III	17 (12.98)	13 (7.34)	​
IV	83 (63.36)	121 (68.36)	​
Prior chemotherapy exposure	​	​	0.062
Cisplatin	5 (3.82)	3 (1.69)	​
Fluoropyrimidines	26 (19.85)	52 (29.38)	​
Gemcitabine	76 (58.02)	112 (63.28)	​
Irinotecan	5 (3.82)	10 (5.65)	​
Nab-paclitaxel	74 (56.49)	114 (64.41)	​
Oxaliplatin	10 (7.63)	10 (5.65)	​
Raltitrexed	0 (0.00)	3 (1.69)	​
Chemotherapy-naïve	50 (38.17)	42 (23.73)	​
The mean number of doses (cycle±SD)	4.54 ± 2.81	5.75 ± 3.50	0.086
The mean dose intensity (mg±SD)	79.41 ± 9.98	221.90 ± 35.52	<0.001

Abbreviations: IRI, non-liposomal irinotecan; nal-IRI, liposomal irinotecan.

As shown in Table 2 and Supplementary Table S1, in the nal-IRI group, the most frequently reported AEs (≥10%) included anemia (22.90%), leucopenia (28.24%), neutropenia (36.64%), thrombocytopenia (26.72%), diarrhea (18.32%), alanine aminotransferase (ALT) increased (17.56%), aspartate aminotransferase (AST) increased (16.03%), hypoalbuminemia (13.74%), and poor appetite (19.85%). In the IRI group, the most common AEs (≥10%) were anemia (28.25%), leucopenia (34.46%), neutropenia (40.11%), thrombocytopenia (22.60%), diarrhea (20.90%), ALT increased (22.03%), AST increased (24.29%), hypoalbuminemia (18.08%), and poor appetite (14.69%).

**TABLE 2:** Summary of hematologic treatment-emergent adverse events in the retrospective study.

Adverse events	nal-IRI (n = 131) n (%)		IRI (n = 177) n (%)	
	Any grade	Grade3-4	Any grade	Grade3-4
Anemia	30 (22.90)	5 (3.82)	50 (28.25)	5 (2.82)
Febrile neutropenia	0 (0.00)	0 (0.00)	0 (0.00)	3 (1.69)
Leucopenia	37 (28.24)	12 (9.16)*	61 (34.46)	6 (3.39)
Neutropenia	48 (36.64)	17 (12.98)	71 (40.11)	31 (17.51)
Thrombocytopenia	35 (26.72)	0 (0.00)	40 (22.60)	5 (2.82)

Abbreviations: IRI, non-liposomal irinotecan; nal-IRI, liposomal irinotecan; *p < 0.05 versus corresponding IRI, group.

For grade 3–4 AEs (≥1% and <10%), the nal-IRI group showed anemia (3.82%), leucopenia (9.16%), neutropenia (12.98%), vomiting (1.53%), fever (1.53%), ALT increased (1.53%), and AST increased (1.53%). The IRI group exhibited anemia (2.82%), febrile neutropenia (1.69%), leucopenia (3.39%), neutropenia (17.51%), thrombocytopenia (2.82%), diarrhea (1.69%), fever (1.13%), ALT increased (3.39%), AST increased (2.26%), and blood bilirubin increased (1.13%). No significant differences were observed in overall AEs between the two groups, either for all grades or grade 3–4 events. However, the incidence of grade 3–4 Leucopenia was significantly higher in the nal-IRI group compared to the IRI group (9.16% vs. 3.39%, p < 0.05).

**FIGURE 2:**
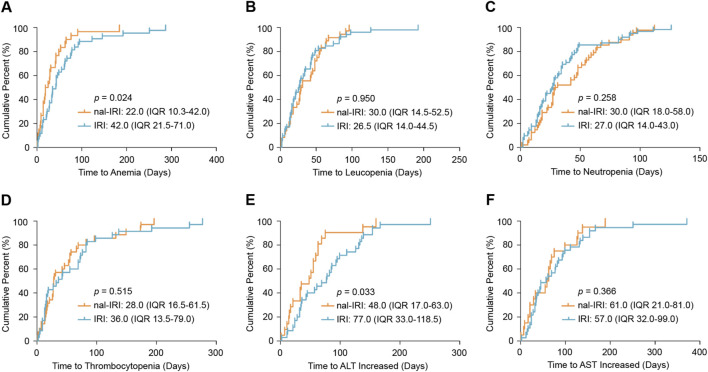
The cumulative distribution curves of the onset time of liposomal and non-liposomal irinotecan -related adverse events. **(A)** Anemia; **(B)** Leucopenia; **(C)** Neutropenia; **(D)** Thrombocytopenia; **(E)** Alanine aminotransferase (ALT) increased; **(F)** Aspartate aminotransferase (AST) increased. Abbreviations: AE, adverse event; ALT, alanine aminotransferase; AST, aspartate aminotransferase; IQR, interquartile range; IRI, non-liposomal irinotecan; nal-IRI, nanoliposomal irinotecan.

Time-to-onset analysis revealed that nal-IRI was associated with earlier development of certain toxicities (Figure 2). The median time to anemia onset was 22.0 days (interquartile range [IQR] 10.3–42.0) for nal-IRI versus 42.0 days (IQR 21.5–71.0) for IRI (p = 0.024). Similarly, ALT increased occurred earlier with nal-IRI (median 48.0 days, IQR 17.0–63.0) compared to IRI (median 77.0 days, IQR 33.0–118.5; p = 0.033). No significant differences were observed in the time-to-onset for leukopenia, neutropenia, thrombocytopenia, or AST increased between the two formulations. These findings suggest that while the overall safety profiles of nal-IRI and IRI are comparable, nal-IRI may lead to earlier onset of hematologic and hepatic toxicities, warranting closer monitoring during the initial treatment cycles.

### 3.2 Demographic characteristics of AE reports, AE profiles and clinical outcomes in the FAERS database

**FIGURE 3:**
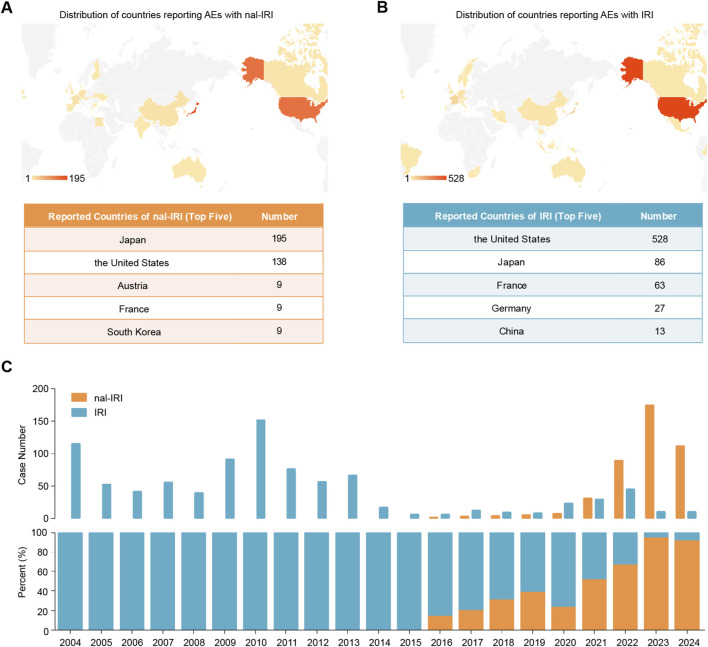
Reporting country and year distribution of liposomal and non-liposomal irinotacan related adverse events reported in the US Food and Drug Administration Adverse Event Reporting System database. **(A)** Reporting country distribution of liposomal irinotecan. **(B)** Reporting country distribution of non-liposomal irinotecan. **(C)** Reporting year distribution of liposomal irinotecan or non-liposomal. The bar chart above displays the annual number of adverse events associated with the two drugs, while the proportional bar chart below shows the proportional distribution of adverse events linked to liposomal and non-liposomal irinotecan formulations each year. Abbreviations: AE, adverse event; IRI, non-liposomal irinotecan; nal-IRI, nanoliposomal irinotecan.

From January 2004 to December 2024, we identified 7,630 AE reports associated with either liposomal or conventional IRI in the FAERS database. After deduplication, 425 reports for nal-IRI and 917 for IRI were included in the analysis (Figure 1). The demographic characteristics revealed significant differences between the two formulations (Table 3). For age distribution, nal-IRI reports were predominantly from patients >65 years (43.29%), followed by unknown age group (28.71%), while IRI reports most frequently lacked age documentation (42.53%), with 35.11% from 18–64 year olds. Both formulations showed similar gender distributions, with slight male predominance (nal-IRI: 50.12%; IRI: 50.38%). Clinical outcomes differed significantly, with nal-IRI showing higher fatal outcomes (43.76% vs. 29.99%) compared to IRI. Primary indications also varied substantially, with nal-IRI primarily reported for pancreatic cancer (72.71%) versus IRI for colorectal cancer (36.75%). Geographical analysis revealed nal-IRI reports were most common in Japan (45.88%) followed by the United States (32.47%), whereas IRI reports predominated in the United States (57.58%) with fewer from Japan (9.38%) (Figure 3). Temporal trends showed distinct patterns: nal-IRI reports emerged in 2016, peaked in 2023 (174 cases, 40.94%), then declined in 2024 (111 cases, 26.12%), while IRI reports were concentrated in 2004–2013, peaking in 2010 (151 cases, 16.47%) with substantially fewer reports after 2014. These findings demonstrate important differences in reporting patterns between the two formulations across multiple demographic and clinical parameters.

**TABLE 3:** Demographic and clinical characteristicsof reports with adverse events caused by liposomal and non-liposomal irinotacan sourced from the US Food and Drug Administration Adverse Event Reporting System database (from 1 January 2004 to 31 September 2024).

Characteristics	Nal-IRI	IRI	p Value
Patients (n)	425	917	​
Age (years)	​	​	<0.0001
<18	7 (1.65)	14 (1.53)	​
18–64	112 (26.35)	322 (35.11)	​
≥65	184 (43.29)	191 (20.83)	​
Unknown	122 (28.71)	390 (42.53)	​
Gender	​	​	0.597
Male	213 (50.12)	462 (50.38)	​
Female	154 (36.24)	313 (34.13)	​
Unknown	58 (13.65)	142 (15.49)	​
Outcome	​	​	<0.0001
Death	186 (43.76)	275 (29.99)	<0.0001
Hospitalization	98 (23.06)	211 (23.01)	0.984
Life-threatening	5 (1.18)	30 (3.27)	0.025
Other	90 (21.18)	233 (25.41)	0.092
Unknown	46 (10.82)	168 (18.32)	0.0005
Indications (top 5)	​	​	​
​	Pancreatic cancer, 309 (72.71)	Colorectal cancer, 337 (36.75)	​
	Oesophageal cancer, 12 (2.82)	Lung cancer, 56 (6.11)	​
	Biliary tract cancers, 10 (2.35)	Pancreatic cancer, 46 (5.02)	​
	Colorectal cancer, 8 (1.88)	Gastric cancer, 34 (3.71)	​
	Sarcoma, 4 (0.94)	Oesophageal cancer, 29 (3.16)	​

Abbreviations: IRI, non-liposomal irinotecan; nal-IRI, liposomal irinotecan.

**FIGURE 4:**
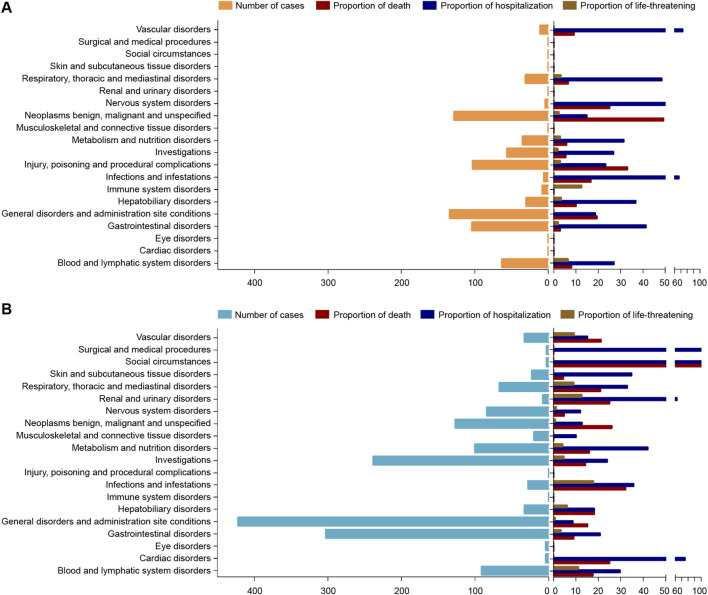
System Organ Class distribution of adverse events associated with liposomal and non-liposomal irinotecan reported in the US Food and Drug Administration Adverse Event Reporting System database (from 1 January 2004 to 31 September 2024). The left bar chart displays the number of reported cases for liposomal irinotecan **(A)** and non-liposomal irinotecan **(B)** across different System Organ Classes. The right bar chart shows the proportion of patient outcomes (death, hospitalization, or life-threatening events) within each System Organ Class for liposomal irinotecan **(A)** and non-liposomal irinotecan **(B)**.

The analysis of SOC distributions revealed distinct AE patterns between nal-IRI and IRI (Figure 4). For nal-IRI, the top five reported SOCs were general disorders and administration site conditions (134 cases), neoplasms benign, malignant and unspecified (128 cases), gastrointestinal disorders (104 cases), injury, poisoning and procedural complications (103 cases), blood and lymphatic system disorders (63 cases). In contrast, IRI showed higher reporting rates for general disorders and administration site conditions (422 cases), gastrointestinal disorders (303 cases), investigations (238 cases), neoplasms benign, malignant and unspecified (127 cases), and metabolism and nutrition disorders (100 cases).

Mortality analysis by SOC demonstrated that nal-IRI-associated deaths primarily involved neoplasms benign, malignant and unspecified (49.22%), injury, poisoning and procedural complications (33.01%), and renal and urinary disorders (25.00%), general disorders and administration site conditions (19.40%), infections and infestations (16.67%). IRI-related mortality was most prominent in social circumstances (100%), Infections and infestations (32.14%), and cardiac disorders (25.00%), renal and urinary disorders (25.00%), respiratory, thoracic and mediastinal disorders (20.90%). Hospitalization rates differed substantially, with nal-IRI showing higher rates for vascular disorders (72.73%), infections and infestations (66.67%), and nervous system disorders (50%), respiratory, thoracic and mediastinal disorders (48.39%), gastrointestinal disorders (41.35%); while IRI demonstrated elevated hospitalization frequencies for social circumstances (100%), surgical and medical procedures (100%), cardiac disorders (75.00%), renal and urinary disorders (62.50%), and and nutrition metabolic disorders (42.00%).

At the PT level (Table 4; Supplementary Table S2), nal-IRI was most commonly associated with malignant neoplasm progression (116 cases), off-label use (90 cases), and death (89 cases), whereas IRI was predominantly linked to death (203 cases), diarrhea (162 cases), and disease progression (131 cases). Signal strength analysis identified particularly strong associations for nal-IRI with cholangitis infective (PRR = 996.70), cholangitis (PRR = 60.77) and malignant neoplasm progression (PRR = 56.63); while IRI showed strongest signals for colon cancer (PRR = 49.88), disease progression (PRR = 22.68) and leukopenia (PRR = 13.89).

**TABLE 4:** Signal strength (PRR) of the top 30 adverse events related to liposomal and non-liposomal irinotacan sourced from the US Food and Drug Administration Adverse Event Reporting System database (from 1 January 2004 to 31 September 2024).

No.	Nal-IRI			IRI		
	PTs	n	PRR	PTs	n	PRR
1	Malignant neoplasm progression	116	56.63	Death	203	3.33
2	Off label use	90	4.64	Diarrhoea	162	5.64
3	Death	89	4.15	Disease progression*	131	22.68
4	Diarrhoea	55	4.63	Vomiting	57	2.43
5	Neutrophil count decreased	30	31.67	Neutropenia	49	6.99
6	Decreased appetite	22	5.03	Dehydration	43	7.52
7	Vomiting	20	2.19	Pyrexia	41	2.71
8	Myelosuppression	20	24.53	Abdominal pain	35	3.60
9	Disease progression*	16	6.57	Colon cancer*	34	49.88
10	Anaemia	16	4.22	Leukopenia	29	13.89
11	Pyrexia	15	2.12	Sepsis*	25	5.31
12	White blood cell count decreased	14	5.66	Haemoglobin decreased	24	5.52
13	Neutropenia	14	5.56	Anaemia	24	3.16
14	Product use issue*	13	3.55	Platelet count decreased	23	4.24
15	Interstitial lung disease	12	13.31	Thrombocytopenia	22	5.40
16	General physical health deterioration	11	5.37	Neuropathy peripheral*	21	3.92
17	Hypokalaemia	9	9.63	Interstitial lung disease	19	7.85
18	Ascites*	9	18.66	Febrile neutropenia	19	7.61
19	Enterocolitis	9	53.21	White blood cell count decreased	17	3.58
20	Cholangitis*	8	60.77	Muscle spasms	16	2.32
21	Metastases to liver*	8	19.88	Neoplasm progression*	15	8.13
22	Cholangitis infective*	7	996.70	Pancreatic carcinoma*	15	8.66
23	Aspartate aminotransferase increased	7	4.54	Hyperhidrosis	14	2.60
24	Febrile neutropenia	7	6.76	Stomatitis	14	3.76
25	Sepsis*	6	3.27	Neutrophil count decreased	13	8.00
26	Pulmonary embolism	6	3.28	Speech disorder*	13	4.29
27	Gastrointestinal haemorrhage*	6	3.02	Malignant neoplasm progression*	13	3.00
28	Intestinal obstruction	6	8.34	Hyponatraemia	12	4.49
29	Haematotoxicity	6	36.93	Dysarthria*	12	6.82
30	Lymphocyte count decreased	5	16.55	Pulmonary embolism	12	3.04
30	Pneumonitis	5	8.83	General physical health deterioration*	12	2.74
30	Pancreatitis*	5	56.63	-	-	-

Abbreviations: IRI, non-liposomal irinotecan; n, the number of adverse events reports; nal-IRI, liposomal irinotecan; PRR, proportional reporting ratio; PT, preferred terms; –, a negative signal.

^*^ Adverse events mentioned in the label.

Notably, we identified several unlabeled AEs for both formulations. For nal-IRI, these included disease progression, product use issue, ascites, cholangitis, metastases to liver, cholangitis infective, sepsis, gastrointestinal haemorrhage, pancreatitis. IRI demonstrated unlabeled signals for disease progression, colon cancer, sepsis, neuropathy peripheral, neoplasm progression, pancreatic carcinoma, speech disorder, malignant neoplasm progression, dysarthria, general physical health deterioration. These findings highlight important differences in the safety profiles between the two formulations, with nal-IRI showing stronger associations with hepatobiliary complications and IRI demonstrating more frequent hematologic and neurologic toxicities.

## 4 Discussion

### 4.1 Safety analysis based on single-center retrospective study: AEs incidence and time-to-onset

Our single-center retrospective study analyzed 308 pancreatic cancer patients, comparing the incidence and time-to-onset of AEs between nal-IRI and IRI in real-world clinical practice. This study complemented the FAERS database analysis and provided more detailed clinical safety data.

The results demonstrated similar overall AE profiles between the two groups, with both primarily exhibiting hematologic toxicities (anemia, neutropenia, leukopenia) and elevated liver enzymes (ALT, AST), consistent with the known toxicity characteristics of irinotecan (Bailly, 2019). However, the nal-IRI group showed a significantly higher incidence of grade 3–4 leukopenia (9.16% vs. 3.39%, p < 0.05), potentially attributable to drug accumulation and prolonged myelosuppressive effects caused by the liposomal carrier. This difference is unlikely to be explained by imbalances in baseline disease severity or prior chemotherapy history, as both cohorts were well-balanced in terms of disease stage and prior treatment exposure. No significant differences were observed in non-hematologic toxicities such as diarrhea and liver function abnormalities.

This study pioneered the systematic comparison of time-to-onset for AEs between the two formulations. Notably, the nal-IRI group demonstrated earlier onset of anemia (22.0 days vs. 42.0 days, p < 0.05) and ALT increased (48.0 days vs. 77.0 days, p < 0.05). This phenomenon may be explained by the unique pharmacokinetic properties of nal-IRI: the liposomal formulation prolongs circulation and primarily confines distribution to the vascular compartment, resulting in higher plasma concentrations (Adiwijaya et al., 2017), which may lead to more rapid bone marrow suppression and liver enzyme elevation. However, this mechanistic interpretation remains hypothesis-generating, as our study did not include direct pharmacokinetic measurements. The proposed association is therefore inferred from established preclinical and clinical pharmacology literature rather than derived from patient-level exposure data.

Clinical implications and monitoring recommendations: These findings highlight the need for increased clinical vigilance regarding early hematologic and hepatic toxicity risks with nal-IRI. Close monitoring of blood counts (particularly white blood cells and hemoglobin) and liver function during initial treatment cycles is recommended to enable timely intervention. The earlier onset of anemia and liver enzyme elevation suggests potential benefits from adjusted supportive care strategies, such as early administration of erythropoiesis-stimulating agents or hepatoprotective medications.

### 4.2 Safety comparison based on FAERS database: demographic characteristics, AE profiles, and clinical outcomes

This study analyzed AE reports of nal-IRI and IRI in the FAERS database from 2004 to 2024, revealing significant differences in demographic characteristics, AE profiles, and clinical outcomes, providing important references for clinical safety evaluation.

AE reports for nal-IRI primarily involved elderly patients (>65 years, 43.29%) and pancreatic cancer patients (72.71%), while IRI reports were more common in 18–64 year-olds (35.11%) and colorectal cancer patients (36.75%). This distribution difference likely reflects their distinct indications: nal-IRI is mainly used for advanced pancreatic cancer (more prevalent in elderly patients) due to its targeting properties, whereas IRI has long been employed as first- and second-line treatment for advanced colorectal cancer. Additionally, nal-IRI reports were concentrated in Japan and the United States (combined 78.35%), with a significant increase after 2016, consistent with its approval timeline (Brown and Blair, 2025) and regional promotion strategies. In contrast, IRI reports peaked around 2010, possibly related to patent expiration (Bailly, 2019) and generic drug availability. Notably, no statistical difference was observed in gender distribution between the two formulations, suggesting minimal gender influence on AE occurrence.

At the SOC level, both formulations primarily showed systemic reactions (e.g., fatigue, fever) and gastrointestinal toxicity (e.g., diarrhea). However, nal-IRI demonstrated stronger signals for hematologic disorders (e.g., neutropenia) and cholangitis-related AEs (e.g., infective cholangitis, PRR = 996.70), potentially attributable to the liposomal carrier’s accumulation characteristics in the hepatobiliary system. IRI more frequently reported metabolic disorders (e.g., dehydration) and peripheral neuropathy, consistent with known toxicity profiles of conventional irinotecan (Bailly, 2019).

Regarding clinical outcomes, nal-IRI showed significantly higher mortality reporting rates (43.76% vs. 29.99%, p < 0.01), though this requires cautious interpretation. The high mortality SOCs for nal-IRI (e.g., malignant neoplasm progression, 49.22%) were largely associated with the inherently poor prognosis of advanced pancreatic cancer rather than direct drug toxicity. In contrast, IRI-related deaths were more likely associated with treatment-related complications such as infections (32.14%) and cardiac events (25.00%).

Both formulations demonstrated unlabeled AE signals. New signals for nal-IRI (e.g., cholangitis, ascites, sepsis) may relate to liposome-induced local inflammation or biliary excretion disorders (Large et al., 2021), while new IRI-reported AEs (e.g., peripheral neuropathy, speech disorder) expand understanding of its neurotoxicity. These signals require further studies to validate causality.

An important source of confounding in this comparative safety assessment arises from the differential disease indications between the two formulations. In the FAERS database, nal-IRI was predominantly used in patients with advanced pancreatic cancer, a population characterized by poor prognosis, high burden of hepatobiliary metastases, and frequent disease-related complications such as cholangitis, malignant biliary obstruction, and cancer-associated mortality. In contrast, conventional IRI was most commonly reported in patients with colorectal cancer, a population with comparatively longer survival and different toxicity susceptibility. These disparities in underlying disease likely contribute substantially to the observed differences in AE profiles, particularly the higher reporting rates of fatal outcomes and hepatobiliary events in the nal-IRI group. While our single-center retrospective cohort partially addresses this confounding by restricting both treatment groups to pancreatic cancer patients, the FAERS analysis inherently lacks adjustment for cancer type, stage, and prognosis. Therefore, the formulation-specific safety signals identified in this study should be interpreted as drug–disease composite effects rather than purely pharmacokinetic toxicity. Future pharmacovigilance studies should consider propensity score matching or restriction-based designs to better isolate formulation-related risks.

When interpreting the disproportionality signals identified in this study, particular caution is warranted for extremely high PRR or ROR values, such as the PRR of 996.70 observed for infective cholangitis with nal-IRI. While such extreme point estimates suggest a strong statistical association, they do not necessarily imply a proportional increase in absolute risk. Several factors may contribute to signal inflation in spontaneous reporting databases. First, infective cholangitis is a rare event in the general FAERS background population, which can disproportionately amplify signal strength metrics even with modest case counts. Second, this adverse event is clinically more common in patients with advanced pancreatic cancer—the primary population receiving nal-IRI—due to frequent biliary stenting, tumor invasion of the biliary tree, and obstructive pathophysiology. Thus, the observed signal likely reflects a combination of drug-related hepatobiliary toxicity and disease-specific susceptibility. Third, relatively low absolute case numbers for infective cholangitis can yield unstable signal estimates. Therefore, these findings should be regarded as hypothesis-generating rather than definitive evidence of excessive risk. Future pharmacoepidemiologic studies using large-scale real-world data with appropriate denominator information and disease control groups are needed to validate these signals and quantify absolute risk.

### 4.3 Study limitations

While this study comprehensively evaluated both irinotecan formulations through real-world retrospective research and FAERS database analysis, several limitations warrant consideration. The FAERS database, as a spontaneous reporting system, has inherent limitations including underreporting and incomplete data (e.g., 42.53% missing age information in IRI group and 28.71% in the nal-IRI group). Such missing data may introduce bias in demographic comparisons and limit the precision of subgroup analyses. However, the primary disproportionality signals (ROR and PRR) are derived from adverse event frequencies rather than demographic variables, which partially mitigates this concern. The single-center retrospective study’s limited sample size (n = 308) and single-institution nature may also affect generalizability. While our findings provide valuable real-world insights, multicenter prospective studies with larger, more diverse patient populations are needed to confirm these observations. Importantly, our retrospective cohort did not include pharmacokinetic sampling; therefore, the proposed mechanistic links between liposomal pharmacokinetics and the observed toxicity patterns are based on literature-derived inference rather than direct exposure–response analysis. In addition, FAERS is subject to inherent geographical reporting bias, as it predominantly collects reports from the United States and countries with well-established FDA-linked pharmacovigilance systems. This limits the generalizability of our findings to regions with different reporting infrastructures or clinical practices. The higher mortality in nal-IRI group (43.76%) may reflect advanced pancreatic cancer characteristics rather than drug toxicity, and we were unable to fully control for treatment regimen variations. Temporal differences in reporting patterns (IRI mainly 2004–2013 vs. nal-IRI post-2016) and evolving medical practices may further complicate direct comparisons. These limitations underscore the need for future multicenter prospective studies with pharmacokinetic monitoring and long-term follow-up to better characterize safety profiles.

An additional methodological consideration pertains to the marked difference in mean dose intensity between the nal-IRI and IRI groups in our retrospective cohort (79.41 ± 9.98 mg vs. 221.90 ± 35.52 mg, p < 0.001). While this disparity may appear to confound direct safety comparisons, it is important to consider that liposomal and non-liposomal irinotecan are pharmacokinetically distinct entities rather than dose-equivalent formulations of the same drug. Preclinical evidence suggests that nal-IRI achieves comparable plasma and intratumoral exposure of the active metabolite SN-38 at fivefold lower doses than conventional IRI (Milano et al., 2022; Barbier et al., 2023). Furthermore, population pharmacokinetic analyses indicate that the toxicity drivers differ between formulations: neutropenia is primarily associated with peak SN-38 concentration (C_max_), which is substantially lower with nal-IRI, whereas diarrhea correlates with total irinotecan C_max_, which is prolonged but not directly dose-linear with nal-IRI (Adiwijaya et al., 2017). These differential exposure–response relationships preclude simple milligram-to-milligram extrapolation. To date, no established dose equivalence between nal-IRI and conventional IRI exists in humans, and given their fundamentally different pharmacological profiles, such equivalence remains uncertain. Therefore, the observed dose intensity difference should be interpreted with caution when comparing safety outcomes between groups. It more likely reflects distinct dosing paradigms for two different therapeutic agents rather than indicating imbalanced treatment intensity. Nonetheless, we acknowledge that residual confounding by dose intensity cannot be fully excluded, and future prospective studies incorporating pharmacokinetic monitoring are warranted to definitively isolate formulation-specific toxicity signals.

## 5 Conclusion

This study systematically compared the safety profiles of nal-IRI and IRI through FAERS database analysis and single-center retrospective research, yielding several key conclusions: (Voutsadakis et al., 2024): Significant differences in hematologic toxicity were observed in real-world settings. The retrospective study confirmed significantly higher incidence of grade 3–4 leukopenia (9.16% vs. 3.39%) and earlier onset of anemia and elevated liver enzymes (median time 20–30 days sooner) with nal-IRI, suggesting distinct mechanisms of myelosuppression and hepatotoxicity requiring enhanced early monitoring. (De With et al., 2023). Formulation properties determined toxicity characteristics and temporal patterns, with nal-IRI’s rapid onset of early AEs (e.g., anemia, ALT increased) consistent with its carrier-mediated drug release kinetics. (Milano et al., 2022). Clinical implications: nal-IRI use warrants vigilance for early hematologic and hepatobiliary toxicity (Fra and mpton, 2020). Population and indication differences substantially shape the AE reporting patterns of the two formulations. In FAERS, nal-IRI was predominantly administered to elderly patients with advanced pancreatic cancer, a population inherently at higher risk for mortality, malignant progression, and hepatobiliary complications. Thus, the high reporting rates of death and cholangitis associated with nal-IRI likely reflect a combination of disease-related baseline risk and formulation-specific hepatobiliary toxicity due to liposomal accumulation. In contrast, IRI was mainly used in colorectal cancer patients and exhibited conventional chemotherapy toxicities such as diarrhea and neutropenia. These findings underscore the need for contextualized safety interpretation and highlight the importance of controlling for disease indication in future comparative pharmacovigilance studies.

## Data Availability

The original contributions presented in the study are included in the article/Supplementary Material, further inquiries can be directed to the corresponding authors.
