# Supplementary material for: Distinct safety profiles of liposomal and conventional irinotecan: insights from clinical experience and real-world data
Source: Front Pharmacol. 2026 Apr 21;17:1745317. doi: 10.3389/fphar.2026.1745317 (PMC13139361; doi:10.3389/fphar.2026.1745317)
Supplement: Supplementary file 1 [file Table1.docx]

Supplementary Material

# Supplementary Tables

**Supplementary Table S1. Summary of non-hematologic treatment-emergent adverse events in the retrospective study.**

| Adverse events | nal-IRI (n=131) n (%) | | IRI (n=177) n (%) | |
| --- | --- | --- | --- | --- |
|  | Any grade | Grade3-4 | Any grade | Grade3-4 |
| Eye disorders |  |  |  |  |
| Watering eyes | 0(0.00) | 0(0.00) | 1(0.56) | 0(0.00) |
| Gastrointestinal disorders | 0(0.00) | 0(0.00) | 0(0.00) | 0(0.00) |
| Abdominal distension | 4(3.05) | 0(0.00) | 3(1.69) | 0(0.00) |
| Abdominal pain | 1(0.76) | 0(0.00) | 4(2.26) | 0(0.00) |
| Constipation | 3(2.29) | 0(0.00) | 5(2.82) | 0(0.00) |
| Diarrhea | 24(18.32) | 0(0.00) | 37(20.90) | 3(1.69) |
| Intestinal obstruction | 1(0.76) | 0(0.00) | 1(0.56) | 0(0.00) |
| Mucositis oral | 0(0.00) | 0(0.00) | 1(0.56) | 0(0.00) |
| Nausea | 3(2.29) | 0(0.00) | 8(4.52) | 0(0.00) |
| Vomiting | 7(5.34) | 2(1.53) | 13(7.34) | 0(0.00) |
| General disorders |  |  |  |  |
| Chills | 0(0.00) | 0(0.00) | 2(1.13) | 0(0.00) |
| Edema limbs | 1(0.76) | 0(0.00) | 0(0.00) | 0(0.00) |
| Fatigue | 4(3.05) | 1(0.76) | 7(3.95) | 1(0.56) |
| Fever | 3(2.29) | 2(1.53) | 4(2.26) | 2(1.13) |
| Pain | 0(0.00) | 0(0.00) | 2(1.13) | 0(0.00) |
| Infectious diseases |  |  |  |  |
| Biliary tract infection | 2(1.53) | 0(0.00) | 1(0.56) | 0(0.00) |
| Conjunctivitis | 0(0.00) | 0(0.00) | 1(0.56) | 0(0.00) |
| Hepatic infection | 0(0.00) | 0(0.00) | 1(0.56) | 1(0.56) |
| Pneumothorax | 0(0.00) | 0(0.00) | 1(0.56) | 0(0.00) |
| Upper respiratory infection | 0(0.00) | 0(0.00) | 1(0.56) | 0(0.00) |
| Urinary tract infection | 0(0.00) | 0(0.00) | 1(0.56) | 0(0.00) |
| Investigations |  |  |  |  |
| Alanine aminotransferase increased | 23(17.56) | 2(1.53) | 39(22.03) | 6(3.39) |
| Aspartate aminotransferase increased | 21(16.03) | 2(1.53) | 43(24.29) | 4(2.26) |
| Blood bilirubin increased | 8(6.11) | 1(0.76) | 8(4.52) | 2(1.13) |
| Metabolism and nutrition disorders |  |  |  |  |
| Hypoalbuminemia | 18(13.74) | 0(0.00) | 32(18.08) | 0(0.00) |
| Poor appetite | 26(19.85) | 0(0.00) | 26(14.69) | 0(0.00) |
| Weight loss | 1(0.76) | 0(0.00) | 3(1.69) | 0(0.00) |
| Neoplasms benign, malignant and unspecified |  |  |  |  |
| Tumor hemorrhage | 1(0.76) | 1(0.76) | 0(0.00) | 0(0.00) |
| Nervous system disorders | 0(0.00) | 0(0.00) | 0(0.00) | 0(0.00) |
| Insomnia | 5(3.82) | 0(0.00) | 2(1.13) | 0(0.00) |
| Overall peripheral sensory neuropathy | 0(0.00) | 0(0.00) | 1(0.56) | 0(0.00) |
| Syncope | 0(0.00) | 0(0.00) | 1(0.56) | 1(0.56) |
| Skin and subcutaneous tissue disorders |  |  |  |  |
| Hyperhidrosis | 0(0.00) | 0(0.00) | 1(0.56) | 0(0.00) |
| Urinary disorders |  |  |  |  |
| Creatinine increased | 1(0.76) | 0(0.00) | 1(0.56) | 0(0.00) |
| Vascular disorders |  |  |  |  |
| Thromboembolic event | 0(0.00) | 0(0.00) | 3(1.69) | 1(0.56) |
| Total | 99(75.57) | 29(22.14) | 145(81.92) | 53(29.94) |

Abbreviations: IRI, non-liposomal irinotecan; nal-IRI, liposomal irinotecan; all p ≥ 0.05.

**Supplementary Table S2. ROR and χ² values of the top 30 adverse events related to liposomal and** **non-liposomal irinotacan sourced from the US Food and Drug Administration Adverse Event Reporting System database (from January 1, 2004 to September 31, 2024).**

| No. | nal-IRI | | | IRI | | |
| --- | --- | --- | --- | --- | --- | --- |
|  | PTs | χ² | ROR(95% CI) | PTs | χ² | ROR(95% CI) |
| 1 | Malignant neoplasm progression | 5697.45 | 74.89(60.06-93.37) | Death | 240.88 | 3.76(3.15-4.50) |
| 2 | Off label use | 221.90 | 5.43(4.24-6.96) | Diarrhoea | 519.69 | 6.43(5.35-7.72) |
| 3 | Death | 202.64 | 4.91(3.86-6.25) | Disease progression* | 2455.12 | 25.92(21.37-31.43) |
| 4 | Diarrhoea | 145.16 | 5.12(3.82-6.86) | Vomiting | 35.30 | 2.50(1.84-3.39) |
| 5 | Neutrophil count decreased | 623.50 | 33.34(21.71-51.22) | Neutropenia | 190.23 | 7.25(5.24-10.03) |
| 6 | Decreased appetite | 57.90 | 5.22(3.30-8.27) | Dehydration | 186.47 | 7.77(5.52-10.94) |
| 7 | Vomiting | 10.70 | 2.24(1.40-3.60) | Pyrexia | 34.30 | 2.77(1.96-3.92) |
| 8 | Myelosuppression | 293.10 | 25.34(14.87-43.17) | Abdominal pain | 54.16 | 3.69(2.56-5.30) |
| 9 | Disease progression* | 70.50 | 6.79(4.12-11.19) | Colon cancer* | 1480.17 | 51.65(36.28-73.53) |
| 10 | Anaemia | 29.05 | 4.33(2.49-7.51) | Leukopenia | 286.61 | 14.25(9.58-21.21) |
| 11 | Pyrexia | 6.14 | 2.16(1.22-3.83) | Sepsis* | 69.46 | 5.41(3.51-8.34) |
| 12 | White blood cell count decreased | 37.81 | 5.78(3.18-10.52) | Haemoglobin decreased | 69.86 | 5.62(3.61-8.76) |
| 13 | Neutropenia | 48.13 | 5.71(3.35-9.73) | Anaemia | 29.13 | 3.21(2.08-4.95) |
| 14 | Product use issue* | 19.67 | 3.63(2.04-6.44) | Platelet count decreased | 39.00 | 4.30(2.66-6.94) |
| 15 | Interstitial lung disease | 113.41 | 13.64(7.49-24.82) | Thrombocytopenia | 74.89 | 5.51(3.61-8.41) |
| 16 | General physical health deterioration | 31.45 | 5.47(2.92-10.25) | Neuropathy peripheral* | 27.72 | 3.97(2.34-6.73) |
| 17 | Hypokalaemia | 45.94 | 9.78(4.63-20.64) | Interstitial lung disease | 77.11 | 7.96(4.69-13.49) |
| 18 | Ascites* | 133.37 | 19.04(9.84-36.87) | Febrile neutropenia | 91.28 | 7.74(4.79-12.50) |
| 19 | Enterocolitis | 206.27 | 53.83(22.27-130.10) | White blood cell count decreased | 25.42 | 3.62(2.17-6.03) |
| 20 | Cholangitis* | 294.99 | 61.63(27.51-138.08) | Muscle spasms | 10.78 | 2.34(1.43-3.84) |
| 21 | Metastases to liver* | 89.53 | 20.15(9.00-45.12) | Neoplasm progression* | 80.68 | 8.24(4.86-13.97) |
| 22 | Cholangitis infective* | 2950.96 | 1006.16(370.10-2735.37) | Pancreatic carcinoma* | 87.51 | 8.78(5.18-14.89) |
| 23 | Aspartate aminotransferase increased | 7.80 | 4.57(1.71-12.24) | Hyperhidrosis | 11.30 | 2.62(1.52-4.53) |
| 24 | Febrile neutropenia | 28.90 | 6.86(3.25-14.47) | Stomatitis | 13.58 | 3.78(1.89-7.59) |
| 25 | Sepsis* | 7.36 | 3.30(1.48-7.40) | Neutrophil count decreased | 66.76 | 8.10(4.58-14.31) |
| 26 | Pulmonary embolism | 7.41 | 3.32(1.48-7.42) | Speech disorder* | 19.56 | 4.32(2.24-8.33) |
| 27 | Gastrointestinal haemorrhage* | 4.91 | 3.04(1.26-7.35) | Malignant neoplasm progression* | 14.07 | 3.02(1.71-5.34) |
| 28 | Intestinal obstruction | 25.40 | 8.43(3.49-20.35) | Hyponatraemia | 21.05 | 4.52(2.34-8.72) |
| 29 | Haematotoxicity | 175.22 | 37.45(16.72-83.87) | Dysarthria* | 44.06 | 6.88(3.69-12.84) |
| 30 | Lymphocyte count decreased | 58.33 | 16.73(6.93-40.42) | Pulmonary embolism | 14.54 | 3.07(1.74-5.43) |
| 30 | Pneumonitis | 20.50 | 8.90(3.32-23.83) | General physical health deterioration* | 10.50 | 2.76(1.52-5.00) |
| 30 | Pancreatitis* | 5697.45 | 4.70(1.95-11.36) | - | - | - |

Abbreviations: CI, confidential interval; IRI, non-liposomal irinotecan; nal-IRI, liposomal irinotecan; ROR, reporting odds ratio; PT, preferred terms; χ², chi-square; –, a negative signal; *, adverse events mentioned in the label.
